# How Emotional Intelligence Influences Cognitive Outcomes Among University Students: The Mediating Role of Relational Engagement During the Covid-19 Pandemic

**DOI:** 10.3389/fpsyg.2021.711439

**Published:** 2021-10-25

**Authors:** Javed Iqbal, Muhammad Azeem Ashraf, Shahnaz Perveen, Naima Qureshi, Zahid Imran, Ning Jin

**Affiliations:** ^1^School of Education, Guangzhou University, Guangzhou, China; ^2^Research Institute of Educational Science, Hunan University, Changsha, China; ^3^Department of Education, The Government Sadiq College Women University Bahawalpur, Bahawalpur, Pakistan; ^4^Division of Education, University of Education, Lahore, Pakistan; ^5^Lahore Business School, The University of Lahore, Lahore, Pakistan

**Keywords:** emotional intelligence, cognitive outcomes, relational engagement, Covid-19 pandemic, China

## Abstract

This study investigated the relationships among emotional intelligence (EI), relational engagement (RE), and cognitive outcomes (COs). A survey questionnaire containing 34 statements was completed by 338 undergraduate students from the four universities of China, with responses recorded on a 7-point Likert-type scale. The relationships were examined using the partial least squares structural equation modeling. The findings showed that EI influenced the COs directly and indirectly during the pandemic. In the forms of self-regulation (SR) and social skills (SS), the high levels of EI improved the COs of the students. Further, the aspects of EI, such as SR, self-awareness (SA), empathy (E), motivation (M), and SS were found to improve the RE of the students. The RE was positively correlated with the COs, indicating its potential for improving critical thinking among university students. Finally, the RE was a key mediator of the relationship between the EI and COs. It is concluded that the students with higher levels of EI and RE may achieve better COs. The implications of the research and suggestions for future studies are also discussed.

## Introduction

The COVID-19 pandemic has reshaped the educational landscape worldwide with measures restricting the transmission of the virus such as lockdowns, working from home, and online education (Iqbal et al., 2021a). However, online learning during such public health crises is associated with the fear of infection, boredom, frustration, and insufficient information (Calandri et al., 2021). The students have been required to manage these stressors, uncertainties, and depression linked to the pandemic (Rubin and Wessely, 2020). Previous research suggested that emotional intelligence (EI) predicts such emotional reactions (Kaya et al., 2017) and contributed to the interest of the present study in investigating the EI and cognitive outcome (COs) during the pandemic.

Emotional intelligence has helped the individuals to cope with the pandemic and is also an antecedent of the COs. EI consists of self-awareness (SA), motivation (M), self-regulation (SR), social skills (SS), and empathy (E) (Jan and Anwar, 2019). However, the exact role of each dimension of EI in improving the COs during the pandemic has not been investigated (Mitrović Veljković et al., 2020). EI has been shown to predict relational engagement (RE), educational achievements, and work-related success, among the other positive COs (Cherry et al., 2018). On the assumption that EI is associated with RE and COs, the present study attempted to understand these relationships in greater depth.

The cognitive outcomes consist of cognitive strategy, motor skills, intellectual skills, verbal information, and attitude. Previous research has demonstrated a significant relationship between critical and creative thinking skills and COs (Yazzie-Mintz and McCormick, 2012). The significant correlations between EI and COs have been noted (Fredricks et al., 2016). Similarly, RE has been found to predict and positively correlate with COs (Cherry et al., 2018). This study, therefore, assumed that the COs are dependent on EI and RE and sought to further investigate the correlations between the three areas.

Relational engagement is a subset of student classroom engagement (Zhoc, 2015) that consists of relationships with teachers and peers. It has been shown to improve student-teacher relationships inside the classroom (Hew et al., 2016). Further, RE appears to positively impact some student learning outcomes across the disciplines, such as class attendance, promotion rates, and graduation rates (Craig et al., 2015). RE has been studied as an apparent teacher press and teacher support (Craik and Lockhart, 1972). However, few studies to date have explored the mediating role of RE in higher education. Thus, the present research assumed that RE mediates the associations between EI and COs and sought to further investigate this role in the Chinese universities during the pandemic.

While EI has been the focus of considerable research, the current knowledge of the interrelationship of EI, RE, and COs is incomplete. Most studies have explored the role of RE in advanced countries (Kelly and Turner, 2009; Wang and Holcombe, 2010; Cooper, 2014; Zhoc et al., 2018), with its functions relatively underexplored in the context of emerging nations. Additionally, the pandemic has interrupted the efforts of educational institutions, such as universities, to investigate RE and its relationships with EI and COs. Thus far, RE has not been considered as a significant construct in terms of its relationships with EI and COs. On this basis, the present study aimed to highlight the importance of RE as a means of developing new approaches to improve the COs. Within the research framework of this study, the following questions are addressed:

**RQ 1:** How do the dimensions of EI (such as SA, SR, M, E, and SS) influence the COs and RE?
**RQ 2:** How does RE influence the COs?
**RQ 3:** How does RE mediate the relationship between emotional engagement and COs?

## Literature Review

### EI Studies in China

Emotional intelligence in emerging countries has received less attention, although many studies in China have highlighted its positive influence on the academic outcomes of undergraduate students (Zhoc et al., 2018, 2020; Li and Xu, 2019). In addition, Zhoc (2015) explored the role of EI in COs among higher education students. Despite the evidence of its positive impact, the universities are slow to implement the programs and measures that would help to build the EI of the students. Suwannaset (2010) concluded that EI is particularly necessary for international students in the Chinese context, who often experience stress when attempting to adjust to the demands of their new environment.

### Emotional Intelligence

The two main constituents of EI are emotion and intelligence. Emotions refer to the feelings of individuals, which are derived from internal or external states. They act as the sources of energy, conveying certain information about these states and compelling individuals to act accordingly (Zhoc et al., 2018). Emotions are also described as the planned responses to events that involve physiological, experiential, and cognitive aspects. Intelligence allows humans to think, learn, and solve problems efficiently in the workplace and beyond (Mayer et al., 2008; Olson et al., 2019). Zhang et al. (2020) introduced the combined term “EI.” After the term had been established, the theories and models of EI were developed on the aspects, such as EI skills, traits, and mixed models. The EI ability models focus on the psychological capacities that allow individuals to receive information to attain COs (Jan and Anwar, 2019). These models specify the cognitive skills used in EI to solve the problems linked with emotion. The mixed EI models combine cognitive skills with the personality traits, such as optimism, enthusiasm, and self-confidence (Lee et al., 2017; Jan and Anwar, 2019).

The present study used the trait model of EI developed by Boyatzis et al. (2000) and Petrides and Furnham (2001). Furthermore, the three-stream model for classifying EI (Ashkanasy and Daus, 2005) was applied. In this model, stream 1 is based on Mayer and Salovey's (1997) four-branch model of ability, stream 2 includes numerous self- and peer-report measures, while stream 3 consists of additional aspects that do not feature in Mayer and Salovey's model. The present study emphasized the stream 2 approach and focused on the self-report measures.

### Relational Engagement

Relational engagement includes social integration, social belonging, social inclusion, and social involvement. It refers to the sense of attachment of individuals to their peers, teachers, and other people in their educational organization (Hu and Bentler, 1998; Huang, 2021). RE helps individuals to build confidence and to increase their energies once M fails (Bentler and Bonett, 1980; Huang, 2021). The notion of RE with peers, instructors, and the school is also described as an emotional engagement by some scholars (Fredricks et al., 2004; Henseler et al., 2014). However, RE is a term widely used in research in higher education contexts (Vizoso et al., 2018). RE often occurs in learning the situations outside the classroom, such as interactions with academic supervisors, teachers, and campus peers (Bentler and Bonett, 1980; Huang, 2021). This study combines these understandings of the term to focus on the RE in terms of student relationships with teachers and peers during the pandemic.

### Cognitive Outcomes

The COs denote a set of various purposes and perspectives for learning (Trigueros et al., 2019). The COs are referred to the progress in the academic achievements of students. Oriol-Granado et al. (2017) view COs as the intellectual efforts and activities of the students that result from the teaching and learning process. The cognitive learning outcomes are the primary indicators of educational quality (Xu et al., 2020) and can be assessed *via* exams and continuous assessment. The previous studies show that different factors influence the cognitive learning outcomes of the students (Gallego et al., 2016). The current study defines COs as creative and critical thinking skills, global understanding, and problem-solving skills.

#### Development of Hypotheses and Theoretical Framework

**Figure 1:**
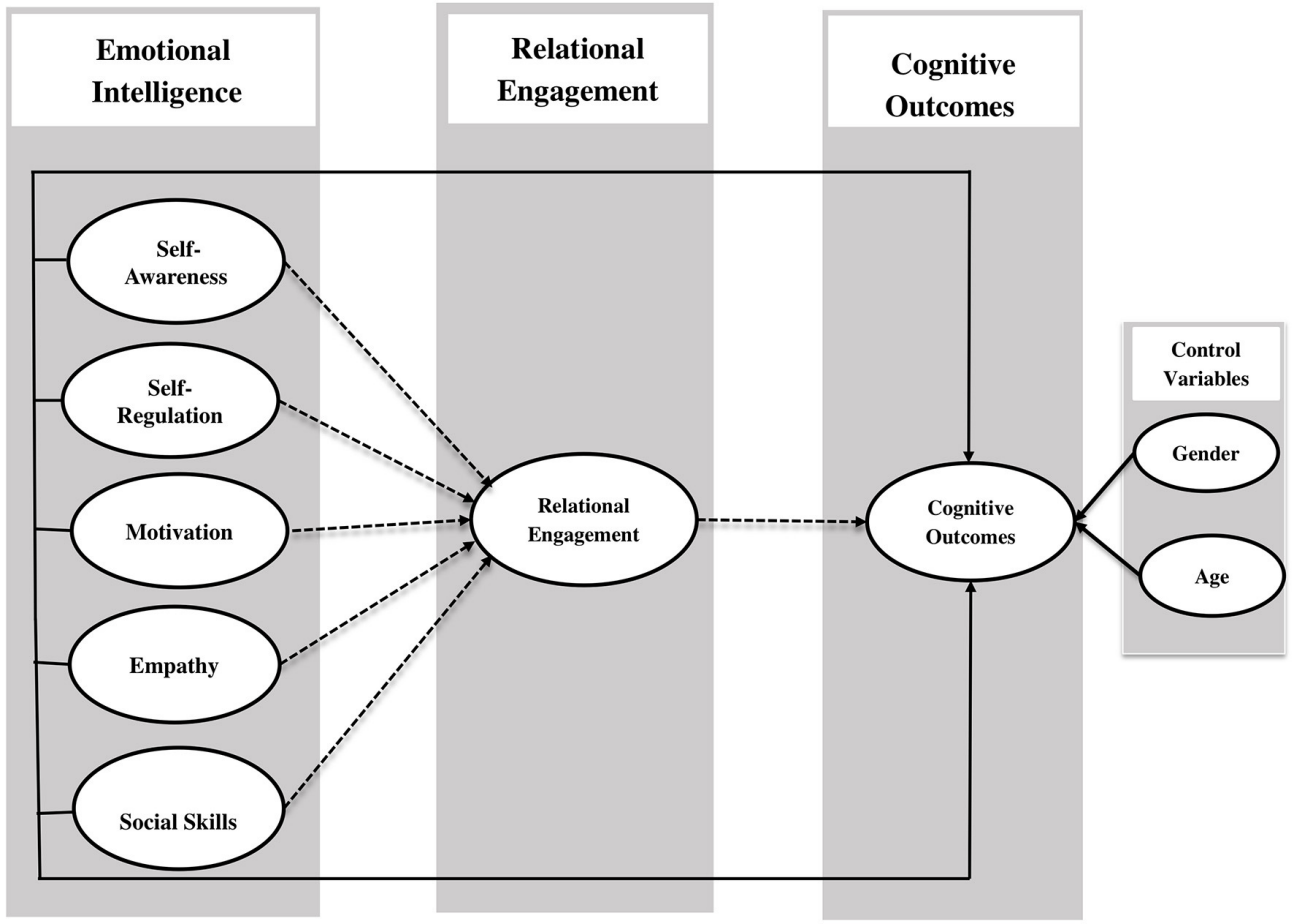
Theoretical framework.

The model described in this study aimed to illuminate how emotional engagement affects the COs through RE. As previously mentioned, EI and RE have occupied a considerable amount of research attention. EI both predicts the COs and exerts many variable effects on them (Zhoc, 2015; Cherry et al., 2018). The theory of student involvement contends that EI may play a significant role in the COs (Lei et al., 2018). In the educational environment, RE impacts university students and may improve the COs. Thus, the current study contends that RE affects the relationship between EI and COs, and aims to investigate how this occurs. To do so, it empirically analyzes these connections and highlights the influence of EI on the COs through RE. The study also helps to clarify the previous literature by explaining the role of EI in affecting the RE that leads to the COs. Furthermore, it is recognized that the students can attain better outcomes through learning EI and better RE. While COs have been defined as the ability to solve complex problems and think critically and creatively (Xu et al., 2020), they might also be evaluated in relation to the EI of teachers. Finally, it has been found that the EI of students can help their RE to improve their COs (Zhoc et al., 2018). On this conceptual basis, the following research model was proposed (Figure 1).

#### Emotional Intelligence and Cognitive Outcomes

The positive relationships between the EI dimensions of SA, SR, M, SS, and E, and COs are well-established (Estrada et al., 2021). Multiple studies have previously identified the roles of different dimensions of EI and their effects on the COs, confirming the positive relationship between the two constructs (Li, 2009; Seifert et al., 2010; Estrada et al., 2021). A student involvement theory similarly supports the importance of EI in achieving cognitive learning outcomes (Zhoc, 2015). Furthermore, strong evidence exists to support the claims that EI and RE are positively associated with positive learning outcomes among university students. Therefore, a positive relationship between the EI and COs is predicted by the following hypotheses:

**H1.1**. Self-awareness positively influences COs.
**H1.2**. Self-regulation positively influences COs.
**H1.3**. Motivation positively influences the COs.
**H1.4**. Empathy positively influences the COs.
**H1.5**. Social skills positively influence the COs.

#### Emotional Intelligence and Relational Engagement

The literature confirms that the students with high EI are more engaged in the classroom *via* the relationships they develop with the teachers and peers (Zhoc et al., 2020). There is a clear pattern of research suggesting that EI positively influences RE (Olivier et al., 2019). Zhoc (2015) explored the relationship between EI and RE among the undergraduates in Hong Kong and found a significant relationship between the two. Thomas and Allen (2021) study of how the EI of undergraduates affected their RE and indicated a significant effect of the former on the latter. Research conducted by Merino-Tejedor et al. (2018) on the Spanish undergraduates recorded similar results, confirming those of earlier studies. Hence, the assumption of a significant relationship between EI and RE informs the following hypotheses:

**H2.1**. Self-awareness positively influences RE.
**H2.2**. Self-regulation positively influences RE.
**H2.3**. Motivation positively influences RE.
**H2.4**. Empathy positively influences RE.
**H2.5**. Social skills positively influence RE.

#### Relational Engagement and Cognitive Outcomes

The theory of student involvement states that RE—especially the relationships of students with the teachers and peers—helps to improve the COs (Zhoc et al., 2018). Lincoln (2009) detected a positive association between student engagement and cognitive learning outcomes, while Davis (2003) found that the teacher-student relationship positively affected such outcomes. Similarly, Roksa and Kilgo (2017) noted that the diverse interactions of students in the places of learning were a helpful way to develop positive COs. However, the association has not been confirmed in all the fields of education: Post et al. (2019) directed the researchers to investigate the relationship of RE and COs in additional subjects besides that of engineering, which featured in their study. Based on the previous findings, the assumption of a positive relationship between the RE and COs informs the following hypothesis:

**H3**. Relational engagement positively influences the COs.

#### EI and COs: The Mediating Role of RE

Emotional intelligence predicts the academic outcomes among the students in university (Gupta and Suman, 2017; Lei et al., 2018). RE is an antecedent of the cognitive learning outcomes (Zhoc, 2015) and is positively correlated with the COs of the students. Hong et al. (2018) explored the direct interaction among the emotional and cognitive interests of the students, behaviors of the teachers, and student engagement. The results suggested that EI and RE are positive predictors of cognitive interests. Thus, in line with this three-way association, we predicted a positive and significant relationship among the EI, RE, and COs. This investigation into the mediating role of RE in the relationship between EI and COs hypothesized the relationship as follows:

**H4.1** RE mediates the relationship between EI (SA) and COs.
**H4.2** RE mediates the relationship between EI (SR) and COs.
**H4.3** RE mediates the relationship between EI (M) and COs.
**H4.4** RE mediates the relationship between EI (E) and COs.
**H4.5** RE mediates the relationship between EI (SS) and COs.

## Methodology

This study was carried out in the context of higher education in China, where little research to date has been conducted. It was motivated by the need to investigate how the strategies of China for coping with the COVID-19 pandemic may have impacted the relationship among the EI, RE, and the COs of university students in the country.

### Questionnaire Design

**Figure 2:**
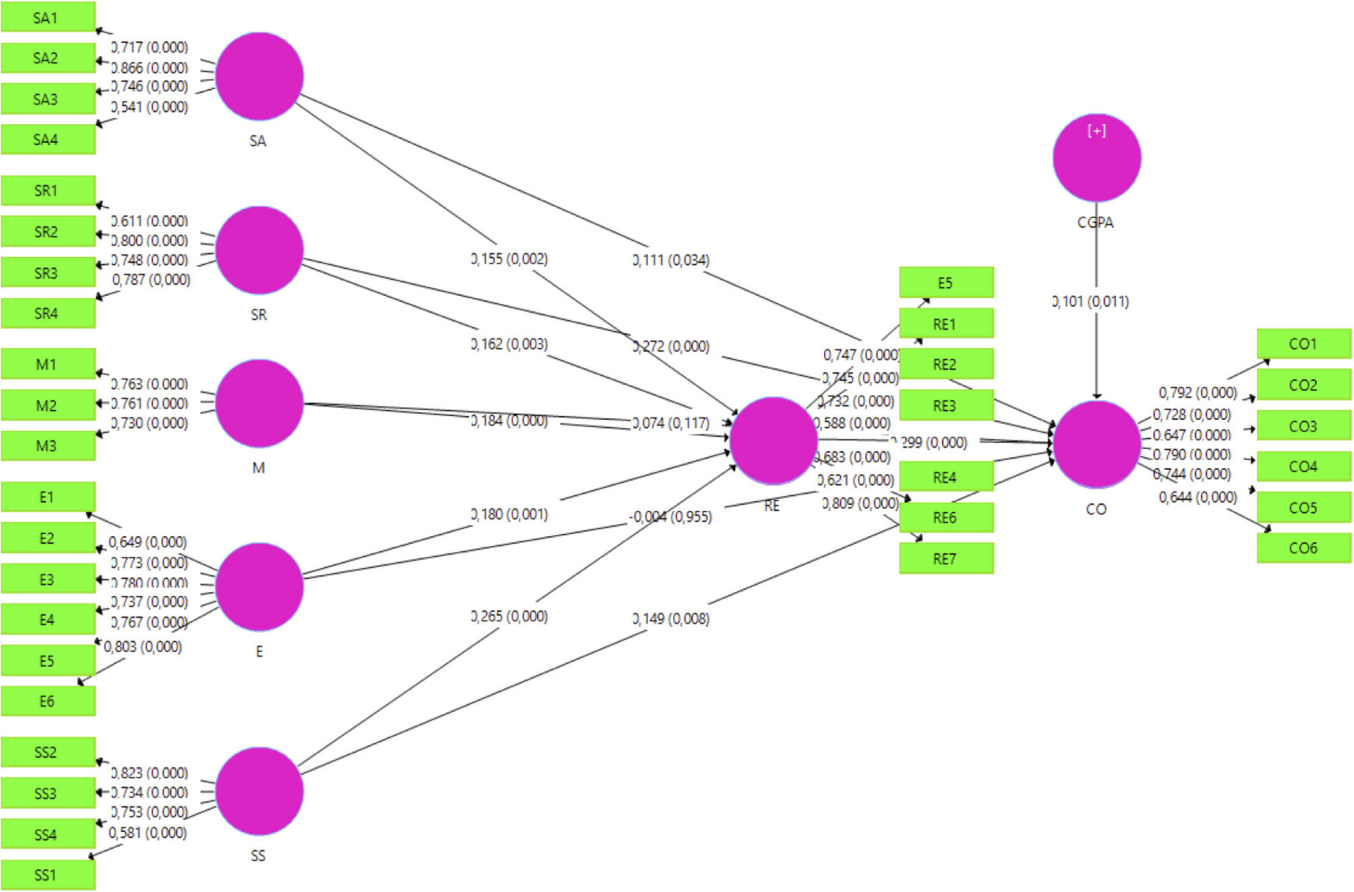
Determines the theoretical constructs with *R*^2^ values.

Data were collected using a survey questionnaire which contained 34 items graded on a 7-point Likert scale (1 = strongly disagree; 7 = strongly agree). The items were adapted from the previous studies related to EI, RE, and COs (Perera and DiGiacomo, 2013; Lau, 2017; Zhoc et al., 2020). EI was measured by the responses to 21 statements on the subtopics of SA, SR, M, E, and SS. The COs and RE were covered by six and seven statements, respectively. The questionnaire was piloted with 20 participants with similar characteristics to the main sample to ensure its validity and reliability, and feedback from this was used to revise a few of the items to ensure they were comprehensible to all the participants. **Table 2** and Figure 2 display the questionnaire and factor loadings for each item.

### Measures

#### Self-Awareness

The four statements related to the SA were adapted from Zhoc et al. (2020) and Perera and DiGiacomo (2013). The examples of these items included “I am able to identify my emotions in different situations” and “I find it easy to express how I feel in different scenarios.” The Cronbach's alpha value for SA was 0.702 (**Table 2**).

#### Self-Regulation

The four statements related to the SR drew on the study of Zhoc et al. (2020). They included sentences, such as “I am able to control my overthinking” and “I concentrate on a pleasant activity when I am feeling low.” The Cronbach's alpha value for SR was 0.731 (**Table 2**).

#### Motivation

The three items exploring M were also developed from the study of Zhoc et al. (2020), with responses again graded on a 7-point Likert-type scale (1 = strongly disagree to 7 = strongly agree). The examples of statements were “I am determined in achieving goals despite obstacles and setbacks” and “I learn to do better next time.” The Cronbach's alpha value for M was 0.714 (**Table 2**).

#### Empathy

The six items related to the construct of E were developed from those provided in Zhoc et al. (2020). The example statements were “My friends can trust me with their secrets” and “I am supportive of the people going through difficult situations.” The Cronbach's alpha value for E was 0.846 (**Table 2**).

#### Social Skills

The four statements were designed to measure the social skill levels and were again based on Zhoc et al. (2020). The example items were as follows: “It is easy for me to make friends” and “Others can depend on me.” The Cronbach's alpha value for SS was 0.705 (**Table 2**).

#### Relational Engagement

The seven statements covered RE and were based on the study of Zhoc et al. (2020) and Lau (2017). They included “I have a close friend(s) in my class” and “I enjoy working with my classmates on different activities.” The Cronbach's alpha value for RE was 0.83 (**Table 2**).

#### Cognitive Outcomes

Finally, the six statements drawn from Zhoc et al. (2020) focused on the COs. Example statements were “I am dealing with unfamiliar problems” and “I am developing in-depth knowledge in my areas of study.” The Cronbach's alpha value for COs was 0.82 (**Table 2**).

### Sampling and Data Collection

Prior to the data collection, the study was approved by the Institutional Review Board of Hunan University, China. The participants of this study were selected using a convenience sampling strategy that has the advantage of being easily applied (Rasool et al., 2020). We selected the participants from students in undergraduate and postgraduate courses at the six universities in China. Pseudonyms were applied to the participating institutions. Participation in the study was entirely voluntary and informed consent was sought before the questionnaires were distributed. All the participants were provided with detailed information about the aims and objectives of the research and informed that the collected data would be used for research purposes only. In total, 400 questionnaires were distributed and completed by 382 participants, with a response rate of 95.5%. Of these, 44 questionnaires were incomplete or filled incorrectly. Thus, the data were gathered from a total sample of 338 respondents (as shown in Table 1).

**Table 1:** Demographics.

**Characteristics**	**Categories**	**Frequency (n)**	**Percentage (%)**
Gender	Male	185	54.7
	Female	153	45.3
	Total	338	100.0
Background	Rural	135	39.9
	Urban	203	60.1
	Total	338	100.0
Sector	Public	102	30.2
	Private	236	69.8
	Total	338	100.0
Field of study	Social science	169	50.0
	Business sciences	67	19.8
	Pure sciences	102	30.2
	Total	338	100.0

## Results

### Confirmatory Factor Analysis

In the study, confirmatory factor analysis (CFA) was used, owing to its close alignment with the structural equation modeling (SEM). The convergent and discriminant validity of each construct was determined through CFA to ensure the fit of the entire model. Some items were removed during this process to achieve the required levels for the scale. The 0.70 threshold value for data reliability (Hair et al., 2019) was met for all the subscales (as shown in Table 2). Although the threshold for factor loading was set at 0.60, a loading of 0.50 is considered acceptable if the average variance extracted (AVE) exceeds 0.50 (Iqbal et al., 2021b). Table 2 indicates that the results of these tests supported the reliability and validity of the scale.

**Table 2:** Convergent validity and reliability.

**Constructs**	**Loading**	**CR**	**rho_A**	**CR**	**AVE**
Self-awareness		0.702	0.754	0.814	0.529
SA1	0.717				
SA2	0.866				
SA3	0.746				
SA4	0.541				
Self-regulation		0.731	0.759	0.828	0.548
SR1	0.611				
SR2	0.800				
SR3	0.748				
SR4	0.541				
Motivation		0.714	0.714	0.796	0.565
M1	0.763				
M2	0.761				
M3	0.730				
Empathy		0.846	0.850	0.887	0.567
E1	0.649				
E2	0.773				
E3	0.780				
E4	0.737				
E5	0.767				
E6	0.803				
Social skills		0.705	0.737	0.817	0.530
SS1	0.581				
SS2	0.823				
SS3	0.734				
SS4	0.753				
Relational engagement		0.830	0.835	0.874	0.500
RE1	0.745				
RE2	0.732				
RE3	0.588				
RE4	0.683				
RE5	0.747				
RE6	0.621				
RE7	809				
Cognitive outcomes		0.820	0.834	0.87	0.528
CO1	0.792				
CO2	0.728				
CO3	0.647				
CO4	0.790				
CO5	0.744				
CO6	0.644				

A heterotrait-monotrait (HTMT) analysis (Henseler et al., 2015) was used to assess the discriminant validity; this is viewed as more accurate than the earlier approach by Fornell and Larcker (1981) and Rasool et al. (2021). An HTMT value higher than 0.90 indicates insufficient levels of discriminant validity (Hair et al., 2019). Table 3 presents the HTMT value for each construct, none of which exceeded the 0.90 thresholds.

**Table 3:** Discriminant validity.

**Constructs**	**CGPA**	**CO**	**E**	**M**	**RE**	**SA**	**SR**	**SS**
CGPA	1,000							
Cognitive outcomes	0.135	0.727						
Empathy	−0.005	0.455	0.753					
Motivation	−0.025	0.407	0.438	0.751				
Relational engagement	0.111	0.617	0.566	0.478	0.707			
Self-awareness	−0.076	0.438	0.578	0.407	0.506	0.727		
Self-regulation	0.041	0.553	0.394	0.353	0.479	0.323	0.74	
Social skills	0.001	0.531	0.572	0.355	0.583	0.45	0.492	0.728

### Descriptive Statistics

The descriptive statistics for the study are presented in Table 4. For all the responses, the range of mean values is from 4.852 to 5.386, while the range of SD falls within 1.09377–1.29637 (as shown in Table 4).

**Table 4:** Descriptive analysis.

**Subscales**	***N***	**Minimum**	**Maximum**	**Mean**	**Std. Deviation**
Self-awareness	338	1.00	7.00	5.278	1.25198
Self-regulation	338	1.00	7.00	4.852	1.29637
Motivation	338	1.00	7.00	4.946	1.09377
Empathy	338	1.00	7.00	5.460	1.15574
Social skills	338	1.00	7.00	5.187	1.25567
Relational engagement	338	1.00	7.00	5.386	1.12313
Cognitive outcomes	338	1.00	7.00	5.110	1.18965

### Regression Analysis

Bootstrapping (5,000 iterations) using SmartPLS-SEM 3.2.2 (SmartPLS GmbH, Bönningstedt, Germany) was performed to examine the relationships among the variables in the theoretical model (Hair et al., 2019). We also used the partial least squares (PLS), a variance-based structural equation modeling technique (VB-SEM), to measure the reliability and validity of the conceptual variables (the simultaneous appraisal of the measurement model) and analyze the underlying relationships hypothesized among the constructs (the structural model—Sarstedt et al., 2017).

Table 5 presents the direct effects of the components of EI (SR, SA, M, E, and SS) on the COs of the students. It shows that the SA had a significantly positive influence on COs (*β* = 0.111, *p* < 0.05), which confirmed H1.1. Additionally, the effect of SR on COs (*β* = 0.272, *p* < 0.05), supported H1.2. However, M had no significant positive influence on COs (*β* = 0.074, *p* > 0.05), so H1.3 was not supported. Similarly, E carried no significant influence on CO (*β* = −0.004, *p* > 0.05), meaning H1.4 was not confirmed. SS showed a significantly positive influence on CO (*β* = 0.149, *p* < 0.05), thus supporting H1.5.

**Table 5:** Direct relations.

**Direct relations**	**Estimations**	**Mean**	**SD**	**T statistics**	***P*-values**
SA -> CO	0.111	0.113	0.053	2.121	0.034
SR -> CO	0.272	0.272	0.055	4.903	0.000
M -> CO	0.074	0.076	0.047	1.568	0.117
E -> CO	−0.004	−0.007	0.062	0.056	0.955
SS -> CO	0.149	0.152	0.056	2.647	0.008
SA -> RE	0.155	0.155	0.049	3.144	0.002
SR -> RE	0.162	0.163	0.054	3.017	0.003
M -> RE	0.184	0.185	0.046	3.997	0.000
E -> RE	0.180	0.183	0.055	3.259	0.001
SS -> RE	0.265	0.263	0.062	4.260	0.000
RE -> CO	0.299	0.299	0.058	5.132	0.000
CGPA -> CO	0.101	0.101	0.039	2.556	0.011

*SA, self-awareness; SR, self-regulation; M, motivation; E, empathy; SS, social skills; RE, relational engagement; CO, cognitive outcomes*.

Self-awareness positively influenced RE to a significant level (*β* = 0.155, *p* < 0.05), thus supporting hypothesis H2.1. Furthermore, SR positively affected the RE (*β* = 0.162, *p* < 0.05), upholding H2.2. M was found to positively influence the RE (*β* = 0.184, p < 0.05), confirming the corresponding hypothesis (H2.3), and E positively affected RE (*β* = 0.180, *p* < 0.05), supporting H4.4. SS also significantly influenced the RE (*β* = 0.265, *p* < 0.05), thereby verifying H2.5. RE exerted a positive influence on CO (*β* = 0.299, *p* < 0.05), supporting H2.5, and a similar positive effect was detected for RE on CO. We also measured the effect of a control variable CGPA on CO, detecting a significant and positive relationship (as shown in Table 5 and Figure 2).

To investigate the mediating effects of RE, the study first examined the indirect effects of EI on COs (as shown in Table 6). Table 6 shows the indirect effects of SA (*β* = 0.047, *p* < 0.05) and SR (*β* = 0.049, *p* < 0.05) on the COs. It also breaks down the EI components into the effects of M (*β* = 0.055, *p* < 0.000), E (*β* = 0.054, *p* < 0.05), and SS (*β* = 0.079, *p* < 0.05) on the COs. The mediating effect of RE on the relationship between EI and COs can thus be inferred. To determine the extent of mediation, the changes in the effect sizes of SA, SR, M, E, and SS on the CO were also measured. The complete effects of SA, SR, M, E, and SS (*β* = 0.111, *p* < 0.05; *β* = 0.272, *p* < 0.005; *β* = 0.074, *p* < 0.05; *β* = −0.004, *p* > 0.05; and *β* = 0.0149, *p* > 0.05, respectively) varied in significance (Table 6), indicating that SA, SR, M, E, and SS retained a significant positive relationship with the COs through the mediation of RE, while E and M did not. Thus, the relationship between the COs and the EI components of SA, SR, and SS was partly mediated by RE. On the other hand, the relationship between M and E and COs was fully mediated by RE. Hence, hypotheses 4.1–4.4 were upheld. Figure 2 presents the theoretical constructs, with *R*^2^ values provided.

**Table 6:** Indirect relations.

**Indirect relations**	**Estimations**	**Mean**	**SD**	**T statistics**	***P*-values**
SA -> RE -> CO	0.047	0.046	0.018	2.628	0.009
SR -> RE -> CO	0.049	0.05	0.022	2.254	0.024
M -> RE -> CO	0.055	0.055	0.017	3.257	0.001
E -> RE -> CO	0.054	0.054	0.019	2.773	0.006
SS -> RE -> CO	0.079	0.078	0.023	3.465	0.001

## Discussion

The present study analyzed the impact of EI on the COs through RE during the COVID-19 pandemic among university undergraduate students in China. The theoretical model developed for this study was tested and revised as a result of the analysis. The majority of the previous study was carried out in the advanced countries (Junco et al., 2011; Ravizza et al., 2014), with very few research studies conducted in the emerging nations (Gregory et al., 2014). There is also a dearth of research into higher education and its essential role in improving the COs of students during the COVID-19 pandemic in China. To the best of the knowledge of the authors, the present study was among the first to explore the effect of EI on the COs in the Chinese universities, especially considering RE as a mediator.

The investigation of the study of the direct relationship between the EI and COs confirmed that three out of five dimensions of EI (SA, SR, and SS) significantly influenced the COs, supporting our intuitions in the hypotheses H1.1, H1.2, and H1.5. The previous studies have shown that these skills have a significant positive relationship with the COs (Bentler and Bonett, 1980; Li, 2009; Van Schaaijk et al., 2020). Charoensukmongkol and Phungsoonthorn's (2020a) research into the impact of EI on cognitive processes among University of Malaga undergraduates (*N* = 178) concluded that the higher levels of EI were strongly predictive of COs. The current study found that only the dimensions of M and E were not significantly associated with the COs, meaning that the hypotheses H1.3 and H1.4 were not upheld. A possible reason for this finding is that COVID 19 measures (i.e., lockdowns and online learning) made it more difficult for the students to motivate themselves or show empathetic behavior toward others but did not impact their cognitive achievements. It is plausible to suggest that the curriculum does not currently address the developmental needs of the students in the area of EI and requires some revision.

In terms of its investigation into the positive and direct impacts of EI on RE, the study underlined that EI (SA, M, SR, SS, and E) is directly and positively linked with RE, supporting hypotheses H2.1–H2.5. This confirms the results of the study carried out by Charoensukmongkol and Phungsoonthorn (2020b). Similarly, Hair et al. (2011) analysis of 84 students found that EI and RE occurred naturally in the university environment. Therefore, it may be concluded that the high EI of students enhances their RE in the university.

A direct, positive relationship between the RE and COs was confirmed, upholding H3. This corroborates the previous studies showing that the RE of the students positively affects the COs (e.g., Hair et al., 2011). It also validates Huang (2021), who measured the effects of RE on active and collaborative learning, COs, and student-faculty interaction, finding that RE positively affects the COs. Thus, the study concluded that high RE predicted the COs of students during the COVID-19 pandemic.

Finally, the study also measured how RE mediated the relationships between EI (SA, M, SR, SS, and E) and COs. The results confirmed that RE mediated the association of all the components of EI (SA, SR, M, E, and SS) with COs, thus supporting H4.1–H4.5. They also validate the research of Rönkk and Evermann (2013), who found that EI, in tandem with RE, enhanced the COs. Moreover, our findings corroborate those of Iqbal and Qureshi (2021), who reported an association between the EI and COs; they also confirm the importance of teachers in enhancing the cognitive processes of students (Prafitriyani et al., 2019). Overall, our study underlines that the RE of the students with teachers and peers is a key mediator of the association between the EI and COs.

## Conclusions

The model designed for this study drew on the insights of previous literature and the theory of student involvement. The findings have valuable implications for both educational practitioners and researchers. They highlight the need to attend to the relationships of EI, RE, and COs among the students in the context of China. In particular, SA, SR, and SS were found to have a direct, significant, and positive effect on the COs, which were also directly influenced by M and E, although not at a significant level. RE also had a direct, significant, and positive influence on the COs, mediating the relationship between these and the EI of students.

Four main conclusions can be drawn from the results of this study. First, EI can be confidently associated with and predicts the cognitive achievements of undergraduates; EI may have helped the students in this study to develop their critical and analytical abilities during the pandemic. Second, the high levels of EI increase forms of RE, such as the student relationships with teachers and peers in university. Third, the high levels of RE improved the COs during the COVID-19 pandemic. Finally, the critical role of RE in mediating between the EI and COs indicates its importance to student achievement during the pandemic.

## Implications, Limitations, and Future Research

### Implications

The practical implications of the study for enhancing the COs of students should be noted. First, the teachers should emphasize the RE of students and engage them in various intellectual activities to increase this (e.g., encouraging discussions, asking students to justify their ideas, and building good relationships with the teachers and peers). Second, the teachers must identify the students with lower levels of EI and teach the strategies for boosting their skills in this area, by exploring each of the key components (SA, M, SR, etc.) in turn. Third, the curriculum designers must include the content related to EI, which would benefit the students exposed to demanding situations such as the COVID-19 pandemic. Finally, since the relationships with teachers and peers create a positive impact on the COs, guidance should be provided to the students on how such relationships can be established and maintained. This would be likely to enhance the academic learning outcomes during conditions that resemble those of the pandemic in the future.

### Limitations and Future Research

The study contains several limitations which may affect the interpretation of its results. First, it was conducted in the Chinese cultural context, exclusively with the students from China, and at a unique point in time (the pandemic). All these factors limit the generalizability of the conclusions: empirical evidence from other countries is also required to confirm the study outcomes. Moreover, the data were collected from undergraduate students from three disciplinary areas (social science, business science, and medical sciences). However, natural science students were omitted from the sample and future research should concentrate on such individuals. Finally, future research efforts might explore the relationship between the EI and COs, considering study habits or cognitive engagement as mediating variables.

## Data Availability Statement

The data supporting the conclusions of this article will be made available by the authors, upon reasonable request.

## Ethics Statement

This study was approved by Institutional Review Board of Research Institute of Educational Science, Hunan University. The patients/participants provided their written informed consent to participate in this study.

## Author Contributions

JI and MA: conceptualization. JI: formal analysis and software. JI, MA, and NQ: methodology. JI, ZI, and SP: resources and preparation of the original draft. MA, NQ, and NJ: review of draft and editing. All authors have read and agreed to the published version of the manuscript.

## Funding

This research was supported by the National Natural Science Foundation of China (The Research Fund for International Young Scientists. Grant no. 71950410624). Opinions reflect those of the authors and do not necessarily reflect those of the grant agency.

## Conflict of Interest

The authors declare that the research was conducted in the absence of any commercial or financial relationships that could be construed as a potential conflict of interest.

## Publisher's Note

All claims expressed in this article are solely those of the authors and do not necessarily represent those of their affiliated organizations, or those of the publisher, the editors and the reviewers. Any product that may be evaluated in this article, or claim that may be made by its manufacturer, is not guaranteed or endorsed by the publisher.

## References

[B1] Ashkanasy N. M. Daus C. S. (2005). Rumors of the death of emotional intelligence in organizational behavior are vastly exaggerated. J. Organiz. Behav. 26, 441–452. 10.1002/job.320

[B2] Bentler P. M. Bonett D. G. (1980). Significance tests and goodness of fit in the analysis of covariance structures. Psychol. Bullet. 88:588. 10.1037/0033-2909.88.3.588

[B3] Boyatzis R. E. Goleman D. Rhee K. (2000). Clustering competence in emotional intelligence: insights from the Emotional Competence Inventory (ECI)s, in Handbook of Emotional Intelligence, eds Bar-On R. Parker J. D. A. (San Francisco, CA: Jossey-Bass), 343–362.

[B4] Calandri E. Graziano F. Begotti T. Cattelino E. Gattino S. Rollero C.. (2021). Adjustment to COVID-19 lockdown among Italian University students: the role of concerns, change in peer and family relationships and in learning skills, emotional, and academic self-efficacy on depressive symptoms. Front. Psychol. 12:643088. 10.3389/fpsyg.2021.643088 34489777 PMC8416775

[B5] Charoensukmongkol P. Phungsoonthorn T. (2020a). The effectiveness of supervisor support in lessening perceived uncertainties and emotional exhaustion of university employees during the COVID-19 crisis: the constraining role of organizational intransigence. J. General Psychol. 148, 431–450. 10.1080/00221309.2020.1795613 32691689

[B6] Charoensukmongkol P. Phungsoonthorn T. (2020b). The interaction effect of crisis communication and social support on the emotional exhaustion of university employees during the COVID-19 Crisis. Int. J. Business. Comm. 2020, 1–8. 10.1177/2329488420953188

[B7] Cherry M. G. Fletcher I. Berridge D. O'Sullivan H. (2018). Do doctors' attachment styles and emotional intelligence influence patients' emotional expressions in primary care consultations? An exploratory study using multilevel analysis. Patient Educat. Couns. 101, 659–664. 10.1016/j.pec.2017.10.017 29102062

[B8] Cooper K. S. (2014). Eliciting engagement in the high school classroom: A mixed-methods examination of teaching practices. Ameri. Edu. Res. J. 51, 363–402. 10.3102/0002831213507973

[B9] Craig C. S. Greene W. H. Versaci A. (2015). E-word of mouth: early predictor of audience engagement: how pre-release “e-WOM” drives box-office outcomes of movies. J. Advertis. Res. 55, 62–72. 10.2501/JAR-55-1-062-072

[B10] Craik F. I. Lockhart R. S. (1972). Levels of processing: A framework for memory research. J. Verb. Learn. Verb. Behav. 11, 671–684. 10.1016/S0022-5371(72)80001-X

[B11] Davis H. A. (2003). Conceptualizing the role and influence of student-teacher relationships on children's social and cognitive development. Edu. Psychol. 38, 207–234. 10.1207/S15326985EP3804_2

[B12] Estrada M. Monferrer D. Rodríguez A. Moliner M. A. (2021). Does emotional intelligence influence academic performance? The role of compassion and engagement in education for sustainable development. Sustainability 13:1721. 10.3390/su13041721

[B13] Fornell C. Larcker D. F. (1981). Evaluating structural equation models with unobservable variables and measurement error. J. Market. Res. 18, 39–50. 10.1177/002224378101800104

[B14] Fredricks J. A. Blumenfeld P. C. Paris A. H. (2004). School engagement: Potential of the concept, state of the evidence. Rev. Educat. Res. 74, 59–109. 10.3102/00346543074001059

[B15] Fredricks J. A. Filsecker M. Lawson M. A. (2016). Student engagement, context, and adjustment: addressing definitional, measurement, and methodological issues. Learn. Instruct. 43, 1–4. 10.1016/j.learninstruc.2016.02.002

[B16] Gallego J. Aguilar-Parra J. M. Cangas A. J. Rosado A. Langer A. (2016). Efecto de intervenciones mente/cuerpo sobre los niveles de ansiedad, estrés y depresión en futuros docentes de educación primaria: un estudio controlado. Rev. Psicodidáctica 21, 87–101. 10.1387/RevPsicodidact.13256

[B17] Gregory P. Gregory K. Eddy E. (2014). The instructional network: using facebook to enhance undergraduate mathematics instruction. J. Computers Math. Sci. Teach. 33, 5–26. Available online at: https://www.learntechlib.org/primary/p/42123/

[B18] Gupta M. Suman. (2017). Effect of type of school, gender, and emotional intelligence on academic achievement of secondary school students: An analytical study. ZENITH Int. J. Multidisc. Res. 7, 67–81. Available online at: https://www.indianjournals.com/ijor.aspx?target=ijor:zijmr&volume=7&issue=2&article=008

[B19] Hair J. F. Ringle C. M. Sarstedt M. (2011). PLS-SEM: indeed a silver bullet. J. Market. Theo. Pract. 19, 139–152. 10.2753/MTP1069-6679190202

[B20] Hair J. F. Risher J. J. Sarstedt M. Ringle C. M. (2019). When to use and how to report the results of PLS-SEM. Euro. Bus. Rev. 31, 2–24. 10.1108/EBR-11-2018-0203

[B21] Henseler J. Dijkstra T. K. Sarstedt M. Ringle C. M. Diamantopoulos A. Straub D.. (2014). Common beliefs and reality about PLS: Comments on Rönkkö and Evermann 2013. Organiz. Res. Meths. 172, 182–209. 10.1177/1094428114526928

[B22] Henseler J. Ringle C. M. Sarstedt M. (2015). A new criterion for assessing discriminant validity in variance-based structural equation modeling. J. Acad. Mark. Sci. 43, 115–135. 10.1007/s11747-014-0403-8

[B23] Hew K. F. Huang B. Chu K. W. S. Chiu D. K. (2016). Engaging Asian students through game mechanics: findings from two experiment studies. Comps. Educat. 92, 221–236. 10.1016/j.compedu.2015.10.010

[B24] Hong Q. N. Gonzalez-Reyes A. Pluye P. (2018). Improving the usefulness of a tool for appraising the quality of qualitative, quantitative and mixed methods studies, the Mixed Methods Appraisal Tool (MMAT). J. Evalu. Clin. Pract. 24, 459–467. 10.1111/jep.12884 29464873

[B25] Hu L. T. Bentler P. M. (1998). Fit indices in covariance structure modeling: Sensitivity to underparameterized model misspecification. Psycholog. Meths. 3:424. 10.1037/1082-989X.3.4.424

[B26] Huang C. H. (2021). Using PLS-SEM Model to explore the influencing factors of learning satisfaction in blended learning. Educat. Sci. 11:249. 10.3390/educsci11050249

[B27] Iqbal J. Qureshi N. (2021). A cross-cultural evaluation of the psychometric properties of an emotional intelligence scale in the academia of Pakistan. Element. Edu. Online 20, 1296–1307. 10.17051/ilkonline.2021.02.149

[B28] Iqbal J. Qureshi N. Asghar M. Z. (2021b). Psychometric properties analysis of student classroom engagement scale in the academia of Pakistan. J. Arch. Egypt 18, 355–370.

[B29] Iqbal J. Qureshi N. Ashraf M. A. Rasool S. F. Asghar M. Z. (2021a). The effect of emotional intelligence and academic social networking sites on academic performance during the COVID-19 pandemic. Psychol. Res. Behav. Manag. 14, 905–920. 10.2147/PRBM.S316664 34234587 PMC8254613

[B30] Jan S. U. Anwar M. A. (2019). Emotional intelligence, library use and academic achievement of university students. J. Austral. Lib. Info. Assoc. 68, 38–55. 10.1080/24750158.2019.1572482

[B31] Junco R. Heiberger G. Loken E. (2011). The effect of Twitter on college student engagement and grades. J. Comp. Assist. Learn. 27, 119–132. 10.1111/j.1365-2729.2010.00387.x

[B32] Kaya H. Senyuva E. Bodur G. (2017). Developing critical thinking disposition and emotional intelligence of nursing students: a longitudinal research. Nurse Edu. Today 48, 72–77. 10.1016/j.nedt.2016.09.011 27721088

[B33] Kelly S. Turner J. (2009). Rethinking the effects of classroom activity structure on the engagement of low-achieving students. Teach. Coll. Rec. 111, 1665–1692. Available online at: http://www.tcrecord.org/Content.asp?ContentId=15308

[B34] Lau W. W. (2017). Effects of social media usage and social media multitasking on the academic performance of university students. Comps. Hum. Behav. 68, 286–291. 10.1016/j.chb.2016.11.043

[B35] Lee Y. E. Kim E. Park S. Y. (2017). Effect of self-esteem, emotional intelligence and psychological well-being on resilience in nursing students. Child Health Nursing Res. 23, 385–393. 10.4094/chnr.2017.23.3.385

[B36] Lei H. Cui Y. Zhou W. (2018). Relationships between student engagement and academic achievement: A meta-analysis. Social. Behav. Personal. Int. J. 46, 517–528. 10.2224/sbp.7054

[B37] Li C. Xu J. (2019). Trait emotional intelligence and classroom emotions: A positive psychology investigation and intervention among Chinese EFL learners. Front. Psychol. 10:2453. 10.3389/fpsyg.2019.02453 31736840 PMC6834770

[B38] Li X. (2009). Entrepreneurial competencies as an entrepreneurial distinctive: An examination of the competency approach in defining entrepreneurs (Master Thesis). Singapore Management University, Dissertations and Theses Collection (Open Access). Available online at: https://ink.library.smu.edu.sg/etd_coll/14

[B39] Lincoln Y. S. (2009). Ethical Practices in Qualitative Research. The Handbook of Social Research Ethics. Thousand Oaks, CA: SAGE Publications. 10.4135/9781483348971.n10

[B40] Mayer J. D. Salovey P. (1997). What is emotional intelligence. Emotion. Dev. Emotion. Intellig. 3:31.

[B41] Mayer J. D. Salovey P. Caruso D. R. (2008). Emotional intelligence. Am. Psycho. 63, 503–517. 10.1037/0003-066X.63.6.503 18793038

[B42] Merino-Tejedor E. Hontangas P. M. Petrides K. V. (2018). Career adaptability mediates the effect of trait emotional intelligence on academic engagement. Rev. Psicodidáctica 23, 77–85. 10.1016/j.psicoe.2017.10.002

[B43] Mitrović Veljković S. Nešić A. Dudić B. Gregus M. Delić M. Meško M. (2020). Emotional intelligence of engineering students as basis for more successful learning process for industry 4.0. Maths 8:1321. 10.3390/math8081321

[B44] Olivier E. Archambault I. De Clercq M. Galand B. (2019). Student self-efficacy, classroom engagement, and academic achievement: Comparing three theoretical frameworks. J. Youth Adolesc. 48, 326–340. 10.1007/s10964-018-0952-0 30421327

[B45] Olson M. K. Sutton J. Vos S. C. Prestley R. Renshaw S. L. Butts C. T. (2019). Build community before the storm: The National Weather Service's social media engagement. J. Contingen. Crisis Manage. 27, 359–373. 10.1111/1468-5973.12267

[B46] Oriol-Granado X. Mendoza-Lira M. Covarrubias-Apablaza C.-G. Molina-López V. M. (2017). Positive emotions, autonomy support and academic performance of university students: The mediating role of academic engagement and self-efficacy. Rev Psicodidáctica 22, 45–53. 10.1387/RevPsicodidact.14280

[B47] Perera H. N. DiGiacomo M. (2013). The relationship of trait emotional intelligence with academic performance: A meta-analytic review. Learn. Individ. Diffs. 28, 20–33. 10.1016/j.lindif.2013.08.002

[B48] Petrides K. V. Furnham A. (2001). Trait emotional intelligence: Psychometric investigation with reference to established trait taxonomies. Euro. J. Personal. 15, 425–448. 10.1002/per.416

[B49] Post L. S. Guo P. Saab N. Admiraal W. (2019). Effects of remote labs on cognitive, behavioral, and affective learning outcomes in higher education. Comps. Educat. 140:103596. 10.1016/j.compedu.2019.103596

[B50] Prafitriyani S. Magfirah I. Amir N. Irmawati A. Umanailo M. (2019). Influence of emotional intelligence on mathematics learning outcomes of class VII middle school 9 Buru students. Int. J. Scient. Tech. Res. 8, 1490–1494. Available online at: http://www.ijstr.org/finalprint/oct2019/Influence-Of-Emotional-Intelligence-On-Mathematics-Learning-Outcomes-Of-Class-Vii-Middle-School-9-Buru-Students.pdf

[B51] Rasool S. F. Wang M. Tang M. Saeed A. Iqbal J. (2021). How toxic workplace environment effects the employee engagement: the mediating role of organizational support and employee wellbeing. Int. J. Environ. Res. Publ. Health 18:2294. 10.3390/ijerph18052294 33652564 PMC7956351

[B52] Rasool S. F. Wang M. Zhang Y. Samma M. (2020). Sustainable work performance: the roles of workplace violence and occupational stress. Int. J. Environ. Res. Publ. Health 17:912. 10.3390/ijerph17030912 32024195 PMC7037902

[B53] Ravizza S. M. Hambrick D. Z. Fenn K. M. (2014). Non-academic internet use in the classroom is negatively related to classroom learning regardless of intellectual ability. Comp. Educ. 78, 109–114. 10.1016/j.compedu.2014.05.007

[B54] Roksa J. Kilgo C. A. (2017). Engaging with diversity: How positive and negative diversity interactions influence students' cognitive outcomes. J. Higher Edu. 88, 297–222. 10.1080/00221546.2016.1271690

[B55] Rönkk,ö M. Evermann J. (2013). A critical examination of common beliefs about partial least squares path modeling. Org. Res. Meths 16, 425–448. 10.1177/1094428112474693

[B56] Rubin G. J. Wessely S. (2020). The psychological effects of quarantining a city. BMJ 368:m313. 10.1136/bmj.m313 31992552

[B57] Sarstedt M. Ringle C. M. Hair J. F. (2017). Treating Unobserved Heterogeneity in PLS-SEM: A Multi-Method Approach Partial Least Squares Path Modeling. Cham: Springer. 10.1007/978-3-319-64069-3_9

[B58] Seifert T. A. Pascarella E. T. Goodman K. M. Salisbury M. H. Blaich C. F. (2010). Liberal arts colleges and good practices in undergraduate education: Additional evidence. J. Coll. Stud. Develop. 51, 1–22. 10.1353/csd.0.0113

[B59] Suwannaset W. (2010). The experiential pattern of emotional intelligence displayed by international BBA students. HRD J. 1, 42–50. Available online at: http://hrdjournal.buu.ac.th/public/backend/upload/onlinejournal/file/01082016_147001777837785600.pdf

[B60] Thomas C. L. Allen K. (2021). Driving engagement: investigating the influence of emotional intelligence and academic buoyancy on student engagement. J. Further Higher Edu. 45, 107–119. 10.1080/0309877X.2020.1741520

[B61] Trigueros R. Cangas A. J. Aguilar-Parra J. M. Álvarez J. F. García-Más A. (2019). No more bricks in the wall: Adopting healthy lifestyles through physical education classes. Int. J. Env. Res. Publ. Health 16:4860. 10.3390/ijerph16234860 31816835 PMC6926670

[B62] Van Schaaijk A. Noor Baloch A. Thomée S. Frings-Dresen M. Hagberg M. Nieuwenhuijsen K. (2020). Mediating factors for the relationship between stress and work ability over time in young adults. Int. J. Env. Res. Publ. Health 17:2530. 10.3390/ijerph17072530 32272748 PMC7177359

[B63] Vizoso C. Rodríguez C. Arias-Gundín O. (2018). Coping, academic engagement and performance in university students. Higher Edu. Res. Develop. 37, 1515–1529. 10.1080/07294360.2018.150400633119598

[B64] Wang M. T. Holcombe R. (2010). Adolescents' perceptions of school environment, engagement, and academic achievement in middle school. Am. Educat. Res. J. 47, 633–662. 10.3102/0002831209361209

[B65] Xu X. Chen P. Wang J. Feng J. Zhou H. Li X.. (2020). Evolution of the novel coronavirus from the ongoing Wuhan outbreak and modeling of its spike protein for risk of human transmission. Sci. Chi. Life Sci. 63, 457–460. 10.1007/s11427-020-1637-5 32009228 PMC7089049

[B66] Yazzie-Mintz E. McCormick K. (2012). Finding the Humanity in the Data: Understanding, Measuring, and Strengthening Student Engagement Handbook of Research on Student Engagement. Boston, MA: Springer. 10.1007/978-1-4614-2018-7_36

[B67] Zhang Z.ou L. C. Miao J. J. Zhang Y. X. Hwang G. J. Zhu Y. (2020). An individualized intervention approach to improving university students' learning performance and interactive behaviors in a blended learning environment. Interact. Learn. Environ. 28, 231–245. 10.1080/10494820.2019.1636078

[B68] Zhoc C. H. (2015). Study on the Interrelationships Between Emotional Intelligence, Self-Directed Learning and the First Year Student Engagement in the Hong Kong Context [dissertation]. [HKU Theses Online]: HKUTO.

[B69] Zhoc C. H. Chung T. S. King R. B. (2018). Emotional intelligence (EI) and self-directed learning: examining their relation and contribution to better student learning outcomes in higher education. Br. Educat. Res. J. 44, 982–1004. 10.1002/berj.3472

[B70] Zhoc K. C. H. Chung T. S. H. King R. B. (2020). Emotionally intelligent students are more engaged and successful: examining the role of emotional intelligence in higher education. Euro. J. Psychol. Edu. 35, 839–863. 10.1007/s10212-019-00458-0

